# Supplementary material for: Risk assessment of atherosclerotic cardiovascular diseases before statin therapy initiation: Knowledge, attitude, and practice of physicians in Yemen
Source: PLoS One. 2022 May 26;17(5):e0269002. doi: 10.1371/journal.pone.0269002 (PMC9135296; doi:10.1371/journal.pone.0269002)
Supplement: S1 File — (PDF) [file pone.0269002.s001.pdf]

If you consented to participate, please fill the questionnaire below:

RISK ASSESSMENT FOR CARDIOVASCULAR DISEASE BEFORE STATIN THERAPY INITIATION: KNOWLEDGE, ATTITUDES AND PRACTICE QUESTIONNAIRE

A gentle reminder: In this questionnaire, we would like to know your knowledge, attitudes and practice regarding the risk assessment of cardiovascular disease before statin therapy initiation according to the 2018 ACC/AHA guidelines on the management of blood cholesterol.

**SECTION A: Physicians' demographic data**	
**1. Gender**	☐ Male ☐ Female
**2. Age**	……………….
**3. Current position**	☐ Consultant<br>☐ Specialist<br>☐ Resident doctor<br>☐ General practitioner
**4. Department**	☐ Cardiology<br>☐ Nephrology<br>☐ Internal medicine<br>☐ Others (Please specify):………………….
**5. Current Working place**	☐ Private Hospital<br>☐ Govermental hospital<br>☐ Private Clinic<br>☐ Others (please specify):
6. **How many years have you been in practice (experience years)?**	………………….
**7. On average, how many patients you see per day?**	☐ 1-25 ☐ 26-50 ☐ > 50
**8. In the past month, how many times on average did you prescribe statin therapy?**	☐ zero times ☐ 1-25 times ☐ 26-50 times<br>☐ 51-75 times ☐ 76-100 times ☐ > 100

9. Do you follow any clinical practice guideline for cholesterol management in your patients?

☐ Yes ☐ No

9.1 If your answer to the previous question was 'Yes', which of the following guidelines you usually follow in your practice for cholesterol/ dyslipidemia management?

☐ American College of Cardiology/American Heart Association (ACC/AHA) Guideline on the management of blood cholesterol

☐ European Society of Cardiology/European Atherosclerosis Society (ESC/EAS) guideline

☐ National Institute for Health and Care Excellence (NICE) guideline

☐ Others (Please specify):…………….

10. Do you use a risk calculator for cardiovascular risk assessment in your practice?

 Yes No

10.1 If your answer to the previous question was 'Yes', which of the following CV risk calculators you usually use in your practice?

 Framingham General CVD risk calculator
 ACC/AHA 10-year ASCVD Risk Estimator
 SCORE system
 JBS3 risk calculator
 QRISK3 risk calculator
 Others (Please specify):

SECTION B: Guidelines general awareness

Note: **Atherosclerotic cardiovascular disease** (**ASCVD**)—defined as acute coronary syndromes (ACSs), a history of myocardial infarction (MI), stable or unstable angina, coronary or other arterial revascularization, stroke, transient ischemic attack, or peripheral arterial disease presumed to be of atherosclerotic origin

Please tick the best answer that reflects your awareness:

1. Which of the following best described your awareness about the 2018 ACC/AHA guideline?

 I am not aware about it at all
 I am aware of its existence, but not aware of its contents.
 I am aware of some of the content but have not read the summary or the full report.
 I have read the summary.
 I have read the full report.

2. Are you aware of any differences between the 2018 ACC/AHA guideline and the 2013 ACC/AHA guideline on the management of blood cholesterol?

 Yes No

3. Which of the following best described your knowledge about Framingham CVD risk calculator?

 I am not aware of this risk score/ calculator.
 I am aware of its existence, **but not aware of the parameters** used in it for calculating the risk
 I am aware of its existence, and **aware of some of the parameters** used in it for calculating the risk.
 I am aware of its existence, and **aware of all the parameters** used in it for calculating the risk.

4. Which of the following best described your knowledge about 2013 ACC/AHA ASCVD Risk calculator?

 I am not aware of this risk score/ calculator.
 I am aware of its existence, **but not aware of the parameters** used in it for calculating the risk
 I am aware of its existence, and **aware of some of the parameters** used in it for calculating the risk.
 I am aware of its existence, and **aware of all the parameters** used in it for calculating the risk.

5. Are you aware of any differences between the Framingham General CVD risk calculator and the ACC/AHA ASCVD 10-year risk calculator?

 Yes **No**

6. Are you aware of the web version or the downloadable ASCVD 10-year risk calculator?

 Yes **No**

SECTION C: Knowledge

1. For primary prevention of ASCVD, the 2018 ACC/AHA guideline recommends <u>a 10-year risk</u> calculation for:

☐ Individuals Aged 20-39 years
☐ Individuals aged 40-75 years
☐ Individuals aged >75 years
☐ I don't know

2. For primary prevention of ASCVD, the 2018 ACC/AHA guideline recommends a <u>lifetime risk</u> calculation instead of 10-year risk calculation for:

☐ Individuals Aged 20-39 years
☐ Individuals aged 40-75 years
☐ Individuals aged >75 years
☐ I don't know

3. The ACC/AHA stratify individuals according to their 10-year ASCVD risk into:

☐ Low, borderline, intermediate, and high risk
☐ Low, intermediate, high and very high risk
☐ Very low, low, intermediate, high and very high risk
☐ I don't now

4. According to the 2018 ACC/AHA guideline on the management of blood cholesterol, a 40 years old patient with diabetes mellitus is falling into which risk category for future ASCVD Events:

☐ Low risk ☐ Moderate risk ☐ High risk ☐ Very high risk ☐ I don't know

5. According to the 2018 ACC/AHA guideline on the management of blood cholesterol, a 65 years old smoker patient with a history of myocardial infarction (MI) is falling into which risk category for CVD:

☐ Low risk ☐ Moderate risk ☐ High risk ☐ Very high risk ☐ I don't know

6. During cardiovascular risk assessment for primary prevention, presence of chronic inflammatory conditions (such as rheumatoid arthritis, psoriasis, …) enhance the individual ASCVD risk:

☐ Yes ☐ No ☐ I do not know

7. In adults not on lipid-lowering therapy, measurement of a <u>non-fasting plasma lipid profile</u> is effective in estimating ASCVD risk:

☐ Yes ☐ No ☐ I do not know

8. The AHA/ACC ASCVD risk calculator estimates/predicts the risk of what outcome(s)

☐ Hard ASCVD (CHD death, nonfatal MI, fatal or nonfatal stroke)
☐ Expanded ASCVD (CHD death, nonfatal MI, fatal or nonfatal stroke, coronary revascularization)
☐ Total CVD (CHD death, MI, coronary insufficiency, angina, ischemic stroke, hemorrhagic stroke, transient ischemic attack, intermittent claudication, and heart failure)
☐ I do not know

9. The ACC/AHA 10-year ASCVD risk calculator may underestimate risk in:

☐ Patients with higher socioeconomic status
☐ Patients receiving consistent screening and preventive care
☐ Patients with chronic inflammatory diseases
☐ I do not know

10. Coronary Artery Calcium score (CAC) can be useful to refine risk assessment and aid in decision making about statin use, mostly in:
☐ Individuals with 10-year ASCVD risk less than 5%<br>☐ Individuals with 10-year ASCVD risk 7.5% - <20%<br>☐ Individuals with 10-year ASCVD risk ≥ 20%<br>☐ I do not know

Section D: Attitudes

Please read each statement carefully and indicate to what extent you agree or disagree with each of the following statements:

	Strongly disagree	Disagree	Neutral	Agree	Strongly agree
1. CV risk assessment is a vital step for the primary prevention of CVD					
2. CV risk assessment should be made an integral part of clinical practice					
3. CV Risk assessment is important for initiating or delaying statin therapy					
4. Healthcare professionals should take the opportunity of any clinic encounter with an individual to screen for CV risks					
5. All adult patients >40 years old who are free of ASCVD and visiting my clinic should have a complete lipid profile for CV risk assessment.					
6. A 10-year risk calculation should be performed for all my adult patients >40 years old who are free of ASCVD					
7. CV risk calculators are reliable tools to predict cardiovascular risk					

Section E: Risk assessment practices with patients					
**How often do you do each of the following in your practice for primary prevention of CVD?**	**Never**	**Rarely**	**Sometimes**	**Frequently**	**Always**
A. Screening your patients aged 40-75 years for CV risk factors					
B. Recommending a lipid profile for your patients aged 40-75 years for CV risk assessment purposes					
C. Calculating the 10-year ASCVD risk for your patients aged 40-75 years					

Section F: Clinician–Patient risk discussion practices					
**How often do you do each of the following with your patients before starting statin therapy?**	**Never**	**Rarely**	**Sometimes**	**Frequently**	**Always**
3.1 Discussing patient's risk for ASCVD					
3.2 Reviewing patient's lifestyle habits ((e.g., diet, physical activity, weight or body mass index, and tobacco use)					
3.3 Discussing the potential benefits of a healthy lifestyle for risk reduction					
3.4 Discussing the potential benefits of statin therapy for risk reduction					
3.5 Discussing the potential adverse effects of statin therapy					
3.6 Explaining for the patients how and when they should take statin medication					
3.7 Reviewing patient medications to avoid potential statin-drug interactions					
3.8 Discussing the importance of adherence to a healthy lifestyle					
3.9 Discussing the importance of adherence to statin therapy					
3.10 Cost consideration (discuss the ability of the patient to pay for the medication and consider that when prescribing the anti-hyperlipidemic agent)					
